# *Anaplasma phagocytophilum* Encephalitis: A Case Report and Literature Review of Neurologic Manifestations of Anaplasmosis

**DOI:** 10.3390/idr15040035

**Published:** 2023-06-29

**Authors:** Ronin Joshua S. Cosiquien, Nenad Stojiljkovic, Charles W. Nordstrom, Emeka Amadi, Larry Lutwick, Igor Dumic

**Affiliations:** 1 University of Minnesota, Minneapolis, MN 55455, USA; cosiq001@umn.edu; 2 Department of Neurology, Mount Sinai Hospital, New York City, NY 10029, USA; nenadstojiljkovic1997@gmail.com; 3 Department of Hospital Medicine, Mayo Clinic Health System, Eau Claire, WI 54703, USAamadi.emeka@mayo.edu (E.A.); 4 Mayo Clinic Alix College of Medicine and Science, Rochester, MN 55905, USA; 5 PROMED, 9 Babcock St, Unit 3, Brookline, MA 02446, USA

**Keywords:** *Anaplasma phagocytophilum*, tick-borne disease, encephalitis

## Abstract

*Anaplasma phagocytophilum* is an obligate intracellular, Gram-negative pathogen, causative agent of Human Granulocytic Anaplasmosis (HGA). HGA usually manifests as a non-specific febrile illness, accompanied by evidence of leucopenia, thrombocytopenia, and an alteration in liver enzymes. Neurologic manifestations of anaplasmosis are rare and rarely reported. We describe a 62-year-old man who developed encephalitis due to an *Anaplasma phagocytophilum* infection. The patient favorably responded to intravenous doxycycline and recovered without neurological sequela. In the tick endemic area, clinicians should have a high index of suspicion for tick-borne diseases in patients presenting with neurological deficits. A prompt diagnosis and treatment lead to improvements in morbidity and mortality.

## 1. Introduction

*Anaplasma phagocytophilum* is a cause of Human Granulocytic Anaplasmosis (HGA), an emerging tick-borne zoonosis. It is an obligate intracellular Gram-negative bacterium transmitted to humans by a tick vector [1]. In the United States, the tick vector for *A. phagocytophilum* is *Ixodes scapularis* in the northeast and the Midwest, and *Ixodes pacificus* in the Pacific northwest [2]. Over the past two decades, the number of reported cases of HGA (and other tick-borne diseases transmitted by the Ixodes tick) have increased, and this is at least partially explained by climate change, better diagnostic tests, and higher awareness about the infection [3,4].

HGA symptoms start approximately one to two weeks after the initial exposure to an infected tick [5,6,7]. The spectrum of the disease varies. While some patients remain asymptomatic, or present with a mild, non-specific, viral-like illness, some patients develop life-threatening complications [1,2,3,4,5,6,7]. The mortality rate is higher if doxycycline treatment is delayed, patients are immunocompromised, or if infection occurs through a blood transfusion [5].

Neurologic symptoms and complications in HGA are rare [2] and encephalitis as an isolated manifestation of the disease has not been previously reported.

## 2. Case Report

A 62-year-old man was brought in by his family to the emergency department (ED) for an evaluation of acute onset of confusion. The patient was in his usual state of health until the afternoon of the ED evaluation when his wife noted that his speech did not make sense. He was confused and unable to maintain meaningful conversation. The patient reported a mild frontal headache but denied any photophobia, phonophobia, nausea, neck stiffness, vomiting, or abdominal pain. He denied any chest pain, cough, or any muscle or joint pain. The patient lived in rural Wisconsin, USA with his family. He was physically active and spent considerable time in his garden and the woods. The wife recollected that he removed a tick from his skin four weeks earlier. He did not have any rash or any other symptoms around the time of the tick bite. The patient did not have any medical comorbidities and was not taking any prescription medication. He drank alcohol occasionally and did not smoke or use any illicit drugs. He did not report any travel outside of Wisconsin.

A physical examination was notable for normal vital signs, except for a fever of 38.4 degree Celsius. The patient was alert, oriented only to himself, but not time, place, or his surroundings. The neck was supple and there was no rigidity. Meningeal signs were negative. The heart, lung, and abdominal exam was unremarkable. There were no rashes or any evidence of joint inflammation. A complete blood cell count showed normal values of hemoglobin, platelets, and white blood cells. The complete metabolic panel showed normal electrolytes, kidney, and liver function. Glucose was elevated at 390 mg/dl, but the anion gap was normal and blood beta-hydroxybutyrate was normal. Procalcitonin was mildly elevated at 0.22 ng/dl (normal < 0.08 ng/dl) and creatine-phosphokinase (CPK) was mildly elevated at 450 U/L (normal value < 300 U/L). Given the symptoms of headache, confusion, and fever, encephalitis was suspected, and a lumbar puncture (LP) was performed. The cerebrospinal fluid (CSF) was clear, and the Gram stain was negative. A cell count showed 10 nucleated cells, with 50% of lymphocytes, and protein and glucose levels were normal. The CSF and blood were sent for culture and for a meningitis/encephalitis panel (Table 1) which encompasses the broad range of bacterial, viral, and protozoan pathogens that cause invasive CNS disease (performed at Mayo Clinic Laboratories, Rochester, MN, USA). A urine analysis did not show any pyuria, the chest X-ray (CXR) was negative for infiltrates or pleural effusions, and magnetic resonance imaging (MRI) of the brain did not show any evidence of leptomeningeal enhancement or evidence of structural brain abnormalities. The patient was started on an empiric treatment with acyclovir, ceftriaxone, and vancomycin. Despite this, he continued to have a fever and confusion which worsened in the next 24 h. In the meantime, the results of the tick panel testing from the blood reported a positive polymerase chain reaction (PCR) for *A. phagocytophilum* as well as positive IgG with 1: 512 titers (normal < 1:64). The antibodies (IgM and IgG) against *Borrelia burgdorferi* were negative as well as the serology against *Ehrlichia chaffeensis*, *Ehrlichia muris euclairensis* and *Ehrlichia ewingii*/*canis*. *Babesia microti* PCR was negative, and the IgG antibodies were negative as well. A full lists of the tests performed on the blood and CSF are presented in Table 1.

Once these results became available, the patient was loaded with 200 mg of intravenous doxycycline and continued on an intravenous doxycycline treatment of 100 mg every 12 h. Within 24 h of doxycycline initiation, his symptoms improved. He became afebrile and gradually, his mental status returned to baseline. He was discharged home the following day to complete 14 days of therapy with doxycycline for *A. phagocytophilum* encephalitis. At a 3-month follow up with his primary care physician, the patient was without any residual deficit and fully recovered.

## 3. Discussion

Neurologic manifestations of anaplasmosis are less common than in other tick-borne diseases such as ehrlichiosis, Lyme disease, and Powassan virus infection [2,6]. Headache is a common symptom of anaplasmosis, reported in as many as 40% of patients [5]. Central nervous system (CNS) involvement in the form of meningitis and encephalitis is very unusual, and according to some authors less than 1% of patients have CNS involvement during HGA [6]. However, a recent study from Europe demonstrated that up to 20% of patients present with meningitis, which is more than previously reported [8]. Some patients might present with stroke-like symptoms [9], and in some cases with ischemic or hemorrhagic stroke [10,11]. One patient in the literature presented with encephalitis complicated with seizures; however, the patient from that report was co-infected with *Ehrlichia chaffeensis* [12]. Unlike patients with Lyme disease who might present with acute transverse myelitis [13], no such neurological manifestation has been reported in anaplasmosis.

In addition to CNS, the peripheral nerves can be damaged in patients with anaplasmosis. Cases of trigeminal neuralgia [14], brachial plexopathy [15], transient hearing loss [16], and bilateral facial nerve paralysis [17] have been reported. A recent systematic review of published cases on anaplasmosis also reported cases of peripheral neuropathy at the site of the tick bite [5]. The exact pathophysiology and why patients develop neurological manifestations of anaplasmosis is unclear.

*A. phagocytophilum* is pathogenic and infects granulocytes as well as endothelial cells [1,10]. This most likely explains the higher propensity for bacterial superinfection following HGA [5], and why some patients develop a stroke [10], respectively. Thus far, it has not been described that *A. phagocytophilum* infects neurons; however, its presence has been demonstrated in CSF [17]. Increased cytokine production has been described during anaplasmosis and this might contribute to some of the unexplained manifestations [5,18]. The exclusion of co-infection with pathogens that more commonly affect the nervous system such as *E. chaffeensis*, *B. burgdorferi*, and Powassan virus is mandatory [1,2,8]. Recent studies from Europe pointed towards higher rates of meningoencephalitis in patients who are co-infected with anaplasmosis and the tick-borne encephalitis (TBE) virus than in patients with an *A. phagocytophilum* monoinfection [8].

Our patient presented with a headache, fever, encephalopathy, and CSF findings of lymphocytic pleocytosis. Other extensive workups for encephalopathy including other infectious diseases, structural CNS lesions, and metabolic causes have been excluded. Additionally, our patient responded promptly to intravenous doxycycline which further confirms that this indeed was encephalitis due to *A. phagocytophilum*.

CSF findings in anaplasmosis, and other tick-borne diseases transmitted by *Ixodes* ticks usually show lymphocytic pleocytosis, normal glucose, and normal to slightly elevated protein [2]. Our patient had CSF findings that were supportive of this diagnosis. However, CSF might be completely normal, and this finding does not rule out diagnosis [2,6]. While intrathecal antibody production is used for the diagnosis of neuroborreliosis, no commercial tests are available to test for *A. phagocytophilum* PCR from CSF or for CSF antibodies against the pathogen. One study in dogs evaluated the presence of CSF antibodies against *A. phagocytophilum* and concluded that the presence of antibodies is not diagnostic of CSN disease [19].

It is important to highlight that our patient, despite the clinical and laboratory evidence of encephalitis, did not have any MRI changes suggestive of it. A negative brain MRI does not rule out disease since the sensitivity of an MRI for the diagnosis of encephalitis is about 80% [20,21,22]. We performed an MRI without contrast; however, recent studies showed that this is an acceptable modality and that the administration of contrast does not significantly improve sensitivity [20,21,22]. CNS anaplasmosis and encephalitis are rare, and no studies have evaluated the sensitivity of an MRI in these patients. However, it is important to highlight that the sensitivity of an MRI for diagnosis depends on the etiology (for example, it is higher for *Herpes simplex* (HSV) encephalitis than for acute disseminated encephalomyelitis-ADEM) [20,21,22]. While some studies have previously described that the sensitivity of an MRI for the diagnosis of encephalitis was lower in children, recent studies found comparable results in the sensitivity between adults and children [20].

Doxycycline is a drug of choice for the treatment of anaplasmosis [23]. Unlike in patients with Lyme neuroborreliosis, who must be treated differently in a setting of CNS involvement (ceftriaxone rather than doxycycline), the treatment of anaplasmosis is the same-regardless of the organ and organ system involved. Doxycycline has reduced CSF penetration compared to ceftriaxone [24,25] which explains why ceftriaxone is the preferred agent for the treatment of neuroborreliosis. However, all cases with anaplasmosis which had neurological symptoms and signs recovered successfully with the use of doxycycline. Other antibiotics that are active in vitro against *A. phagocytophilum* include levofloxacin and rifampin [25]. However, these agents have neither been evaluated against this pathogen in vivo nor in clinical trials for neurological manifestations.

## 4. Conclusions

As cases of anaplasmosis are becoming more frequent, it has become apparent that some patients, such as the one we report, present with predominantly neurological manifestations. In tick-endemic areas, clinicians should keep HGA in mind in patients who present with encephalitis. Further research is necessary for a better understanding of the neurological complications of anaplasmosis and the pathophysiology behind neural damage.

## Figures and Tables

**Table 1:** Panel of laboratory examinations, and results performed on blood and cerebrospinal fluid (CSF) samples of the patient.

Pathogen/Test	CSF	Blood
Gram stain	Negative	-
Cultures	Negative	Negative
*Herpes simplex virus I and II* PCR	Negative	-
*Varicella Zoster Virus* PCR	Negative	-
*Cytomegalovirus* PCR	Negative	-
*Human parechovirus* PCR	Negative	-
*Human Herpesvirus* 6 PCR	Negative	-
*Epstein Barr Virus* PCR	Negative	-
Enterovirus 71 PCR	Negative	-
Adenovirus PCR	Negative	-
LCM virus IgM and IgG	Negative	-
*West Nile Virus* IgM and IgG Ab	Negative	Negative
*Jamestown Canyon Virus* IgM Ab	Negative	-
St. Louis Encephalitis Virus IgM and IgG Ab	Negative	-
Calif (LaCrosse) Encephalitis Virus IgM and IgG	Negative	-
West Equine Encephalitis Virus IgM and IgG Ab	Negative	-
*Human Immunodeficiency* Virus PCR	-	Negative
East Equine Encephalitis Virus IgM and IgG Ab	Negative	-
Powassan virus IgM, IgG, PRNT	Negative	Negative
*Escherichia Coli K1* PCR	Negative	-
*Neisseria meningitidis* PCR	Negative	-
*Borrelia burgdorferi* PCR, IgM, IgG Ab	Negative	Negative
*Leptospirosis* spp. IgM and IgG	Negative	Negative
IGRA	-	Negative
*Listeria monocytogenes* PCR	Negative	Negative
*Ehrlichia eauclairensis* PCR	-	Negative
*Ehrlichia chaffensis* IgM and IgG	-	Negative
***Anaplasma phagocytophilum* IgG**	**-**	**Positive (1:512)**
*Anaplasma phagocytophilum* IgM	-	Negative
***Anaplasma phagocytophilum* PCR**	**-**	**Positive**
*Borrelia miyamotoi* PCR	-	Negative
*Streptococcus pneumoniae* PCR	Negative	-
*Streptococcus agalactiae* PCR	Negative	Negative
*Haemophilus influenza* PCR	Negative	Negative
VDRL	-	Negative
Babesia microti PCR, IgG	-	Negative
*Cryptococcus neoformans*/*gattii* PCR	Negative	-

Ab—antibodies; CSF—cerebrospinal fluid; IgM—immunoglobulin M; IgG—immunoglobulin G; IGRA—interferon-gamma release assays; PCR—polymerase chain reaction; PRNT—plaque reduction neutralization test; VDRL—venereal disease research laboratory.

## Data Availability

Publically available and upon request by corresponding author.
